# A case study of the development of a valid and pragmatic implementation science measure: the Barriers and Facilitators in Implementation of Task-Sharing Mental Health interventions (BeFITS-MH) measure

**DOI:** 10.1186/s12913-024-11783-6

**Published:** 2024-11-06

**Authors:** Lawrence H. Yang, Judy K. Bass, PhuongThao D Le, Ritika Singh, Dristy Gurung, Paola R. Velasco, Margaux M. Grivel, Ezra Susser, Charles M. Cleland, Rubén Alvarado, Brandon A. Kohrt, Arvin Bhana

**Affiliations:** 1 Department of Social and Behavioral Sciences, New York University School of Global Public Health, 708 Broadway, New York, NY 10003 USA; 2 Department of Mental Health, Johns Hopkins Bloomberg School of Public Health, 615 N. Wolfe Street, Room W1114, Baltimore, MD 21205 USA; 3 Center for Global Mental Health Equity, Department of Psychiatry and Behavioral Health, George Washington University, 2120 L St NW, Washington DC, 20037 USA; 4 Transcultural Psychosocial Organization (TPO) Nepal, Anek Marg, Kathmandu, 44600 Nepal; 5 Institute of Psychiatry, Psychology, and Neuroscience, King’s College London, Denmark Hill, London, SE5 9RS UK; 6 Universidad O’Higgins, Avenida Bernardo O’Higgins 1058, Santiago, Chile; 7 Universidad Católica de Chile, Av. Libertador Bernardo O’Higgins 340, Santiago, Región Metropolitana 8331150 Chile; 8 Universidad de Chile, Avenida Bernardo O’Higgins 1058, Santiago, Chile; 9 Columbia University Mailman School of Public Health, 722 west 168th, New York, NY 10027 USA; 10 New York State Psychiatric Institute, 1051 Riverside Dr, New York, NY 10032 USA; 11 Department of Population Health, New York University Grossman School of Medicine, 180 Madison Avenue, New York, NY 10016 USA; 12 Universidad de Valparaíso, Faculty of Medicine, School of Medicine, Department of Public Health, Center for Interdisciplinary Health Studies (CIESAL), Angamos 655, Viña del Mar, Chile; 13 University of KwaZulu-Natal, Centre for Rural Health, Howard College campus, Mazisi Kunene Road, Glenwood, Durban, South Africa; 14 South African Medical Research Council, Health Systems Research Unit, 491 Peter Mokabe Ridge Rd, Overport, Durban, South Africa; 15 Department of Community Health Sciences, Boston University School of Public Health, 801 Massachusetts Avenue, Suite 431, Massachusetts 02118 Boston, USA

**Keywords:** Task-sharing, Mental Health Services, Implementation science, Measure development, Measure adaptation, Measure validation; Case Study

## Abstract

**Background:**

Few implementation science (IS) measures have been evaluated for validity, reliability and *utility* – the latter referring to whether a measure captures meaningful aspects of implementation contexts. We present a real-world case study of rigorous measure development in IS that assesses Barriers and Facilitators in Implementation of Task-Sharing in Mental Health services (BeFITS-MH), with the objective of offering lessons-learned and a framework to enhance measurement utility.

**Methods:**

We summarize conceptual and empirical work that informed the development of the BeFITS-MH measure, including a description of the Delphi process, detailed translation and local adaptation procedures, and concurrent pilot testing. As validity and reliability are key aspects of measure development, we also report on our process of assessing the measure’s construct validity and utility for the implementation outcomes of acceptability, appropriateness, and feasibility.

**Results:**

Continuous stakeholder involvement and concurrent pilot testing resulted in several adaptations of the BeFITS-MH measure’s structure, scaling, and format to enhance contextual relevance and utility. Adaptations of broad terms such as “program,” “provider type,” and “type of service” were necessary due to the heterogeneous nature of interventions, type of task-sharing providers employed, and clients served across the three global sites. Item selection benefited from the iterative process, enabling identification of relevance of key aspects of identified barriers and facilitators, and what aspects were common across sites. Program implementers’ conceptions of utility regarding the measure’s acceptability, appropriateness, and feasibility clustered across several common categories.

**Conclusions:**

This case study provides a rigorous, multi-step process for developing a pragmatic IS measure. The process and lessons learned will aid in the teaching, practice and research of IS measurement development. The importance of including experiences and knowledge from different types of stakeholders in different global settings was reinforced and resulted in a more globally useful measure while allowing for locally-relevant adaptation. To increase the relevance of the measure it is important to target actionable domains that predict markers of utility (e.g., successful uptake) per program implementers’ preferences. With this case study, we provide a detailed roadmap for others seeking to develop and validate IS measures that maximize local utility and impact.

**Supplementary Information:**

The online version contains supplementary material available at 10.1186/s12913-024-11783-6.

## Background

Most implementation science (IS) measurement development has been done in high-income health system contexts such as the US, UK, Australia, and select European countries [1]. Because of this limited contextual focus, current IS measures tend to be less applicable in low- and middle-income countries (LMICs) with different cultural contexts and health and economic systems. Among the key differences in health care systems between high-, middle-, and low-income countries are the role of insurance and payment mechanisms [2], and for mental health care specifically, in LMICs the limited availability of secondary and tertiary mental health care facilities [3] has resulted in a greater reliance on non-specialist mental health providers (e.g., community health workers, peers) [4]. Although there has been some growth in IS measure development for use in LMICs [5], the widespread use of measures developed specifically for these contexts, as well as pragmatic examples of the process of developing such IS measures, remain limited.

Standards exist for rigorous measure development and evaluation. Key criteria include defining the concepts of interest (i.e., constructs) based on relevant theory (known as “content validity”) and conducting appropriate analytic tests to assess reliability (i.e., whether measures are consistent) and validity (i.e., whether measures assess what they propose to measure) [6]. Many IS measures have been limited by a lack of clarity in theory or conceptual frameworks and heterogeneity in operationalization of relevant concepts. Illuminating this gap, a review found that the majority of IS measures, in addition to showing insufficient content validity, either did not provide sufficient information about, or were unsatisfactory in multiple psychometric properties [7]. In addition, rich and detailed descriptions of the process by which IS measures capture information that is relevant to implementation processes in global contexts also remain lacking.

Furthermore, few IS measures have been fully evaluated in terms of their pragmatic properties. According to Glasgow and Riley [8], important criteria for pragmatic measures include, among others: important to stakeholders, low respondent burden, actionable, sensitive to change, broadly applicable, and can serve as a benchmark. Efforts to establish criteria to evaluate pragmatic properties of IS measures have yielded substantial conceptual clarity and are pushing the field of IS measurement development forward to achieve greater scientific rigor and practical impact [7, 9–11]. Nevertheless, still largely missing in the literature is a detailed account of the process of developing and validating a pragmatic IS measure, including regarding how stakeholders including program implementers are engaged to enhance the measure’s utility, a key property defined as whether a measure and its items account for the meaningful aspects of the implementation contexts (e.g., cultural relevance, environmental resources, and program processes).

### A lack of IS measures for task-sharing mental health services

Despite an increase in the policies for increasing availability of mental health services, the 2020 WHO Mental Health Atlas, which captures available mental health systems and resources on a country-by-country basis, found there remains limited availability of mental health resources in LMICs [3]. Currently there is a global push for the scale up and integration of mental health services to reduce the mental health treatment gap worldwide [12]. The 2018 *Lancet Commission on Mental Health and Sustainable Development Goals* [12], *Grand Challenges in Global Mental Health* [13], and several systematic reviews [4, 14–21] all strongly advocate that effective implementation of task-sharing strategies can help narrow the treatment gap that is particularly prominent in LMICs. Task-sharing involves the formalized redistribution of care typically provided by those with more specialized training (e.g., psychiatrists, psychologists) to individuals, often in the community, with little or no formal training (e.g., community/lay health workers, peer support workers) [22]. A growing number of efficacious task-sharing mental health interventions exist and can take diverse forms, including but not limited to: utilizing primary care workers to detect and/or deliver mental health care [23–25]; training community health workers to administer psychotherapy interventions for people with common mental disorders [25, 26]; and using community-based workers or peers to provide access to medications and rehabilitation services for people with serious mental illness [27, 28].

Despite the expanding evidence base, we lack a robust understanding of the barriers and facilitators that contribute to implementation success and what these look like across diverse task-sharing mental health interventions and contexts, which is needed to fulfill the promise of task-sharing in addressing the mental health treatment gap [29]. The lack of valid and pragmatic IS measures to identify these barriers and facilitators (i.e., ‘implementation determinants’) [30] across settings and task-sharing programs limits the researchers’ and implementers’ ability in understanding and addressing critical factors of implementation success. Accordingly, we present a real-world case study of novel and rigorous measure development in IS with the objective of offering lessons-learned and a framework for future efforts in this area, with a particular focus on adapting and validating tools for global use.

### Case study: process of developing the BeFITS-MH measure for task-sharing in mental health

The overarching goal of this article is to provide a real-world, rigorous example of measurement development in IS. This case study describes the collaborative process of: (a) developing and; (b) enhancing the utility of the *Barriers and Facilitators in Implementation of Task-Sharing in Mental Health* (BeFITS-MH) measure. The BeFITS-MH measure is intended to be a pragmatic, multi-dimensional, multi-stakeholder measure to help program implementers and researchers assess critical, modifiable (i.e., actionable) implementation factors (i.e., ‘barriers and facilitators’) that affect the acceptability, appropriateness, and feasibility of evidence-based task-sharing mental health interventions. This case study presents the process of developing and piloting the BeFITS-MH measure to aid teaching, practice and research by IS researchers and program implementers. The BeFITS-MH measure is being embedded for validation in task-sharing mental health studies in three global settings: (I) an integrated mental health care package for chronic disorders, including HIV, in South Africa (Southern African Research Consortium for Mental health INTegration [SMhINT]) [31]; (II) a team-based, multicomponent approach for first episode psychosis in Chile (OnTrack Chile [OTCH]) [32] ; and (III) integration of mental health services alongside stigma reduction in primary care in Nepal (Reducing Stigma among Healthcare Providers [RESHAPE]) [33]. Table 1 presents further information about the task-sharing mental health interventions being implemented in each site. Specifically, this case study illustrates how to develop a measure that has contextual relevance and utility across diverse task-sharing mental health programs and settings, and how to engage stakeholders in assessing the construct validity and pragmatic utility of implementation outcome measures.

**Table 1:** Summary of the task-sharing mental health interventions of validation sites for the BeFITS-MH measure

Project	Southern African Research Consortium for Mental health INTegration S-MIhNT	OnTrack Chile OTCH	REducing Stigma among HeAlthcare ProvidErs RESHAPE
Country	South Africa	Chile	Nepal
Principal Investigator	Bhana, A.	Alvarado, R.	Kohrt, B.
Task-Sharing Strategy evaluated with Be-FITS-MH	Integrated mental health package that includes task-shared mental health counselling by trained and supervised non-specialists *	Coordinated care for First-Episode Psychosis (FEP) patients comparing: Usual Care arm – standard outpatient clinical care OTCH arm – coordinated services provided by interdisciplinary team, based on interests, needs, and preferences of study participants	Mental health services integrated into primary care using the Mental Health Gap Action Programme - Intervention Guide (mhGAP-IG) training for primary care workers comparing: Intervention as Usual (IAU) arm – Training led by mental health specialists RESHAPE arm – Training co-facilitated by mental health specialists and people with lived experience of mental illness and aspirational figures
**Participant Groups**			
Clients	250 Primary Care Patients	100 FEP Service Users	500 Primary Care Patients
Providers	20 Lay Counselors	30 Team Providers	216 Primary Care Health Workers (108 IAU; 108 RESHAPE)
Supervisors	1 Clinic Psychologist 1 Mental Health Counselor	4 Chile-based Supervisors 3 NYC^a^-based Supervisors	1 MPhil^b^ Psychologist 6 Psychiatrists

*Integrated mental health package created based on the Reach, Effectiveness, Adoption, Implementation, Maintenance (RE-AIM framework and the Consolidated Framework for Implementation Research (CFIR). The package includes mental health literacy of users; training implementation and uptake of mental health screening tool and assessment by primary health care nurses; training lay counsellors in depression counselling; and training and implementation of a community education and detection tool by community health workers at household level

^a^*NYC* New York City

^b^*MPhil * Masters of Philosophy

This case study begins with our comprehensive process to create a new measure, informed by both IS frameworks and empirical work, to operationalize relevant domains of barriers and facilitators in implementing task-sharing mental health interventions. Rather than referring to a case study research design [34] we use the term “case study” here to refer to a rich narrative description (akin to “case reports” or “case examples” as used in other fields) to provide a real-life example of how to evaluate implementation processes and outcomes in global contexts. Next, we describe the collaborative linguistic, cultural, and contextual adaptation processes undertaken concurrently via in-depth pilot testing across the three global settings to ensure that our IS measure is comprehensible, relevant, and useful for each program and context. Finally, we detail key stakeholders’ understanding of and potential indicators for our IS outcomes of interest: acceptability, appropriateness, and feasibility. We argue that the simultaneous adaptation of a complex IS concept (i.e., barriers and facilitators to task-sharing interventions) across programs and global sites, and the engagement of stakeholders in assessing the validity and utility of implementation outcomes, signify an advance from standard measurement approaches and IS measurement approaches more generally, and that an in-depth illustration of this process also serves as a teaching tool for global implementation researchers more broadly.

## Methods

### Process of developing the BeFITS-MH measure

We undertook an extensive process to develop the BeFITS-MH measure. First, we developed a multi-level conceptual model to guide our understanding of the domains of barriers and facilitators associated with task-sharing mental health interventions. Second, we further specified the conceptual model using two data sources: the Shared Research Projects (below) and a systematic review. Based on the results of this model building process, we constructed the initial draft of the BeFITS-MH measure. The measure was revised through expert feedback from a modified Delphi panel. Further refinements of the BeFITS-MH measure were done during the translation and local adaptation stage. Lastly, we conducted concurrent pilot testing procedures to finalize the BeFITS-MH measure within the three study site programs.

#### Theory-driven and empirically-grounded measure development

In developing our theoretical model, we selected the Consolidated Framework for Implementation Research (CFIR) [35, 36], and the Theoretical Domains Framework (TDF) [37, 38], which together allowed us to enumerate and categorize a wide range of potential implementation determinants—i.e., ‘barriers and facilitators’ [39]. In addition, we also drew on Chaudoir et al.’s framework [9], which specifies that implementation outcomes (e.g., acceptability, feasibility, fidelity, reach, adoption) are predicted by implementation factors (i.e. the barriers and facilitators) in five levels: (I) client; (II) provider; (III) innovation (defined as the evidence-based practice or intervention); (IV) organization; and (V) structural. This framework is especially applicable for task-sharing because it includes the characteristics of the providers who are critical in delivering task-sharing mental health interventions.

We applied and iteratively refined our framework of the domains and constructs for the types of barriers and facilitators using data from two parallel studies: (I) the Shared Research Project,^1^ a qualitative study that collected interview data from 71 service users and 63 task-sharing providers from three NIMH-funded collaborative U19 “hubs” that implemented task-sharing mental health interventions in different LMIC sites; and (II) a systematic review synthesizing 37 articles with reported implementation barriers and facilitators of task-sharing mental health interventions in LMICs – including perspectives from both service users and providers [40]. For each data source, trained research assistants independently coded the transcripts (for the Shared Research Project) or included articles (for the systematic review) for the type of implementation factor, and then came together in small groups (separately by data source) to share, combine, and organize their coding using Microsoft Excel. Codes generated from the two data sources were then reviewed and further combined until we reached what we considered to be the most comprehensive codebook of barriers and facilitators in task-sharing mental health interventions.

**Fig. 1:**
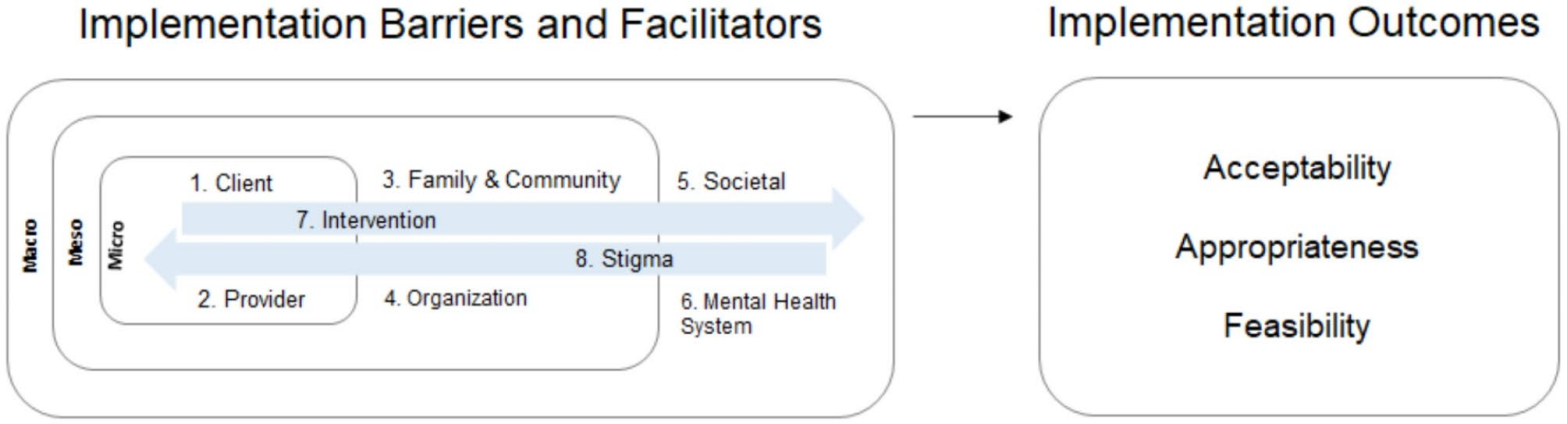
Conceptual model for BeFITS-MH measure

A detailed description of the resulting BeFITS-MH framework and codebook is presented in Le et al. [40]. Briefly, we specify eight domains of task-sharing mental health intervention barriers and facilitators within and across different spheres of influence: (I) client characteristics and (II) provider characteristics in the micro setting; (III) family- and community-related factors and (IV) organizational factors in the meso-level settings; (V) societal and (VI) mental health system-level domains in the macro-level setting; and the (VII) intervention characteristics and (VIII) mental health stigma domains operating across the settings. Figure 1 illustrates the conceptual framework for the BeFITS-MH measure, specifying: (I) the eight domains of barriers and facilitators in implementation of task-sharing mental health interventions, and (II) the three implementation outcomes with which we aim to validate the BeFITS-MH measure: acceptability, appropriateness, and feasibility. We selected these implementation outcomes because they are leading indicators of adoption of evidence-based interventions [41, 42].

#### Initial BeFITS-MH measure

Based on the conceptual framework and the results of the Shared Research Project and the systematic review, we developed an initial version of the BeFITS-MH measure, which contained six subscales and a total of 43 items (6–8 items per subscale), capturing critical aspects of task-sharing mental health implementation barriers and facilitators.

#### Delphi process

To refine the BeFITS-MH measure and to arrive at an expert group consensus of the measure’s core initial domains, format, and structure, we conducted what is known as a ‘modified Delphi process’. Our modifications reflected group sessions that provided opportunities to discuss differences in responses. First, we assembled a ‘Dissemination Panel’ of 19 global experts in implementation of task-sharing mental health interventions and health services research particularly in LMIC settings, including the study co-investigators at the three sites (South Africa, Chile, Nepal). The panel included mostly (17/19, 89%) researchers with global mental health/implementation science expertise spanning various geographical regions and countries: Africa (South Africa, Ethiopia), Asia (Nepal, Viet Nam), South America (Argentina, Chile, Brazil), and the United States (U.S. Latinos, Native Americans). The panel also included two policy makers, one from Lebanon and one from the Pan-American Health Organization (PAHO). We strived to include experts whose work spans across different health systems levels, from policymaking to primary care facilities to engaging with service users and community members.

Over a period of five months, the panel met in three virtual forums (2-hours each), interspersed with two rounds of online questionnaires where panel members were asked to individually provide feedback about different aspects of the BeFITS-MH measure (e.g., the construct and content validity of the domains [subscales], cultural and linguistic appropriateness of the items, hypothesized relationships of subscales to implementation outcomes) (See Additional File 2. BeFITS-MH Delphi Questionnaires – Round 1 and Round 2). All questionnaire responses were compiled and discussed at the following virtual forum. Items with low fit (< 75%) were highlighted and extensively discussed until consensus was reached.

#### Field based translation and local adaptations

Following the Delphi process, we held regular biweekly virtual meetings with the lead BeFITS-MH measure developers and co-investigators from each of the three study sites to translate and locally adapt the BeFITS-MH measure. Within each site, we opted for a group translation process, wherein 2–3 local staff (researchers, clinicians, task-sharing providers, and program implementers) were consulted to jointly translate the measure. This collaborative process has been identified as particularly important for mental health problems and programs, where assessments of emotions and behaviors need to be aligned with local understanding and conceptualizations [43, 44]. Along with the translations, site-specific adaptations included using appropriate terms describing the target mental health problem and task-sharing mental health intervention being implemented within each setting. For example, each site provided project-specific terms used for the [task-sharing] ‘provider’ (e.g., ‘counselor’ in South Africa, ‘team member’ in Chile, and ‘primary health care worker’ in Nepal). Notes regarding how each item was translated and all site-specific adaptations were recorded and discussed during regular biweekly virtual meetings to harmonize the measure across sites and to preserve comprehensiveness of item content (i.e., content validity) to the extent possible.

#### Pilot testing

Piloting of the translated and adapted BeFITS-MH measure was conducted concurrently across the three sites with providers (South Africa 4; Chile 5; Nepal 35) and service users (South Africa 10; Chile 5; Nepal 6). Each site’s research team planned and implemented the pilot data collection in a way that fit their ongoing study. Common across all sites, participants were recruited through established networks, either from ongoing studies (SMhINT, RESHAPE) or in collaboration with healthcare providers (OTCH). Participants were contacted by phone or email (SMhINT, OTCH) or approached in-person during concurrent research activities (RESHAPE). All participants were provided sufficient time to consider their participation and given the opportunity to ask questions before confirming their involvement. As part of the BeFITS piloting process, cognitive interviews were conducted with respondents, including both service users (for the client version) and providers (for the provider version), who were asked to “think aloud” while responding to each item, and to comment on whether items were worded in an understandable way. We further asked whether items were applicable to the specific task-sharing mental health program being implemented and the local setting; this enabled identification of whether the full range of identified barriers and facilitators were used, and in triangulating responses across the three sites, what aspects of barriers and facilitators could be considered core to task-sharing across sites. We also gathered feedback in terms of the project’s preference for the format of the measure (question vs. statement) and the scaling used. The BeFITS-MH research team, comprised of the main tool developers and site co-investigators, discussed the findings during the biweekly virtual meetings, noting site-specific findings as well as commonalities across sites.

### Process of enhancing utility of the BeFITS-MH measure: assessing associations with implementation outcomes

To support later BeFITS-MH validation testing, we describe the process of enhancing the construct validity and utility of the BeFITS-MH measure in assessing three implementation outcomes of interest: acceptability, appropriateness, and feasibility. We did this by pilot testing three brief measures that have been previously used in implementation science research (below) and through stakeholder discussions in each site.

#### Standard measures of implementation outcomes

The three selected measures were the: (a) Acceptability of Intervention Measure (AIM); (b) Intervention Appropriateness Measure (IAM); (c) and Feasibility of Intervention Measure (FIM) [45]. These measures were developed by IS researchers and mental health professionals in the United States, with the vast majority of the developers and the sample of counselors who were part of the development process being Caucasian Americans. The three measures were initially developed for use with mental health counselors in the United States to evaluate the acceptability, appropriateness and feasibility of different treatment options [45]. These measures have been used in English-speaking populations across a range of interventions, including with school-staff for student-wellness programming in England and health care providers providing antenatal alcohol reduction interventions in Australia [46, 47]. More recently, these measures have been used in LMIC settings (Kenya, Tanzania, Botswana, South Africa, and Guatemala) in studies of mental health interventions (depression, anxiety, and alcohol use disorder) including those utilizing task-sharing strategies, HIV services, and medical interventions for genetic disorders and malignant cancers [48–53]. Of note, English language versions of these measures have been used in most settings, with a Swahili version developed in Kenya through translation-back-translation methods [54]. In addition to planning to use these three measures with the task-sharing providers, we explored the potential for using them with the clients and patients who were receiving the task-sharing mental health interventions. Field testing of these three measures with providers and service users was concurrently conducted during the pilot testing of the BeFITS-MH measure (above). After site-specific translation, we made one adaptation to the measures: replacing the term ‘EBP’ with the name of the specific task-sharing program implemented at each site. We then administered the measures to samples of task-sharing providers and service users.

#### Stakeholder feedback sessions

To gain a better understanding from the mental health practitioner and system perspectives regarding the implementation outcomes, we held discussions with local staff in each of the three study countries. These individual and small group conversations were led by site co-investigators using a standard script that included definitions of acceptability, appropriateness, and feasibility, and probes for level-specific indicators for clients, providers, and the setting (Table 2). By indicators we are referring to individual items or programmatic and clinical metrics (like cases seen per month) that can be included in future formal validation testing for the BeFITS-MH measure and are directly related to the measurement of the implementation outcomes. After an introduction of the definitions of the three implementation outcomes, the probes asked the participants to suggest how they think we could best measure these outcomes from the perspective of the people ‘receiving’ the program, the people ‘delivering’ the program, and the locale where the program is being provided.

**Table 2:** Stakeholder feedback definitions and probes

Implementation Outcome	Definitions of Implementation Outcomes	
Acceptability	This is the view that the program is *agreeable and satisfactory* to the people providing the program and to the people receiving the program. This means the program is a good fit for the individuals providing the program and for the people receiving the program.	
Appropriateness	This is the view that the program *fits and is relevant* to the setting, to the people providing the program and to the people receiving the program. This means the program is suitable, compatible, a good fit for the health issue, and/or given the norms and beliefs of the clinic, the people giving the program, or the people receiving the program.	
Feasibility	This is the view that the program *can be successfully used and carried out* by the providers in a given setting. This means the program is possible to do given the resources (such as time, effort, and money), and the circumstances (such as policies, timing).	
**Probes for Programmatic Indicators in Stakeholder Feedback Sessions**		
**Client-level probe**	**Provider-level probe**	**Setting-level probe**
What are your ideas for how we could measure whether *people receiving the program* think the program is acceptable?	What are your ideas for how we could measure whether *people providing the program* think the program is acceptable?	What are your ideas for how we could measure whether *the place where the program is provided* is acceptable?

In Nepal, two small group discussions were held: the first with 3 participants (1 medical officer and 2 senior auxiliary health workers) and the second with 7 participants (4 psychosocial counselors and 3 health systems research staff). In South Africa, one small group discussion was held with 3 program staff (program monitoring and evaluation staff and program implementers). In Chile, information was collected by direct interview of 4 mental health professionals (1 psychologist, 1 occupational therapist, 1 social worker, 1 nurse) working at mental health centers where the task-sharing program is being implemented. Of note, service users were not included in these stakeholder feedback sessions given that it required a clear understanding of the implementation outcomes and the range and types of data available to the research teams, in addition to requiring a deep understanding of the local clinical and health system context as it relates to the implementation of task-sharing mental health interventions. Accordingly, it was determined that engaging with providers and programmatic staff was most appropriate.

The discussions were transcribed and shared in English (for Nepal and Chile) with the full study team. Transcripts were reviewed and coded in Microsoft Excel by the PIs (LHY, JB) and key study team members (PTL, MG) to reach consensus. Transcripts were coded for: (I) each of the three implementation outcomes and; (II) each perspective (client, provider, system). Results were reviewed to identify commonalities and common indicators with a particular eye towards suggesting where differences may be driven by the distinct type of task-sharing program being implemented. Summaries of the stakeholder perspectives around each implementation outcome are presented in the results; recommended programmatic and clinical indicators that can be used for future formal validation testing for BeFITS-MH to enhance its utility are addressed in the Discussion.

## Results

We present major developments and findings of the case study according to our two foci: (I) learnings from the concurrent development of the BeFITS-MH measure in three LMIC settings and (II) learnings from the identification and assessment of key implementation outcomes (acceptability, appropriateness, feasibility) to enhance the utility of later BeFITS-MH validity testing.

### Process of developing the BeFITS-MH measure

The Delphi process resulted in three major adaptations to the BeFITS-MH measure. First, the expert panel agreed that we add a summary or “omnibus” item to each subscale, which is intended to capture the domain’s core concept (i.e., construct). If the omnibus items correlate strongly with the other items within each domain and meaningfully predict implementation outcomes, the omnibus items could potentially be used on their own, reducing the length of the measure and increasing its pragmatic utility. Second, we added a domain on stigma. During the Delphi group discussions, several members highlighted the salience of stigma in task-sharing mental health programs in LMICs and the lack of existing measures to capture stigma-related barriers and facilitators. Three study investigators (LHY, BK, PTL) worked with other experts to develop items for the stigma subscale, which included items that assessed: (a) attitudes of the clients, (b) attitudes about the clients, and (c) provider’s (stigma) experience. Third, the panel came to a consensus to make some of the items optional, a process that we continued in the following steps (below). This was an effort to enhance contextual relevance and to reduce respondent burden. We recognized that some factors, such as cultural/ethnic/caste backgrounds (Item 4.6), are not relevant in certain projects or settings (below). Additionally, there are constructs that are important to assess in the implementation of the intervention in general but were identified as not being specific to the task-sharing strategy itself; items that fell into this grouping were consolidated into a domain called ‘Program Fit’. The study team agreed to make the ‘Program Fit’ and ‘Stigma’ domains optional.

Linguistic translation and cultural and contextual adaptation, along with the pilot testing procedures, each of which took place concurrently in the three sites, led to several important adaptations of the BeFITS-MH measure. We highlight three main findings, regarding: (I) localization; (II) scaling and phrasing; and (III) item selection (i.e., rating of items’ relevance/applicability by site).

A key element to the BeFITS-MH measure was the project-specific adaptations (i.e., “localization”) of broad terms used to refer to aspects of the task-sharing mental health intervention, such as “program,” “provider type,” and “type of service.” This adaptation was necessary due to the heterogeneous nature of the task-sharing mental health interventions, the type of task-sharing providers employed, and the clients served across the three global sites. We included an introductory statement at the beginning of the measure to situate the respondent to the context of their task-sharing mental health intervention. The terms in square brackets ([ ]) were replaced by project-specific terms (see Table 3), allowing for better localization of the task-sharing mental health intervention.

The purpose of this survey is to ask you some questions about your experience participating in [PROGRAM], which involves [TYPE OF SERVICE] delivered by [PROVIDER TYPE] to help with [TARGET PROBLEM].

**Table 3:** A localization of key task sharing for mental health intervention terms

Key Terms	SMhINT (South Africa)		OTCH (Chile)		RESHAPE (Nepal)
Program	Counseling services		OnTrack Chile program		Mental health services in primary care
Client	Patients		User		Patients
Provider type	Counselors		Team of providers		Primary health care workers
Type of service	Depression, anxiety, and adherence counseling		Community care of treatment for first episode psychosis		Treatment for mental health conditions, specifically depression, psychosis, anxiety, and alcohol use disorder
Target problem	Depression, anxiety, and adherence problem		First episode of psychosis		Mental health problems
B Localization of key BeFITS-MH terms					
**SMhINT** **(South Africa)**		**OTCH** **(Chile)**		**RESHAPE** **(Nepal)**	
**Item #1**: The purpose of this survey is to ask you some questions about your experience participating in ***[program]***, which involves ***[type of services]*** delivered by ***[provider type]*** to help with ***[target problem]***.					
The purpose of this *short* survey is to ask you some questions about your experience participating in ***counselling services*** delivered by the ***counsellor*** to help with ***depression***,*** anxiety and adherence problems***.		The purpose of this survey is to ask you some questions about your experience participating in ***OnTrack Chile***, which includes *different care* delivered by ***a team of providers*** where *you are seen for ****the diagnosis of first episode psychosis***.		The purpose of this survey is to ask you some questions about your experience participating in ***a mental health service program***. *When we ask about a**** primary care provider***, *we are asking your opinions about the type of provider who is currently/has recently been providing you with the mental health service* to help with ***problems such as depression***,*** anxiety***,*** alcohol use***,*** or severe mental health problems***.	
**Item #2**: Overall, how satisfied/content are the ***[clients]*** with ***[provider type]*** providing the ***[type of service]***?					
Overall, how satisfied are ***patients*** with ***counselors*** providing a ***depression***,*** anxiety and adherence counseling service***?		Overall, how satisfied are ***users*** with ***the providers*** of the center where you work and ***the OnTrack Chile program*** that is offered?		Overall, how satisfied are the ***patients*** with ***primary health workers*** providing ***treatment for mental health conditions such as depression***,*** psychosis***,*** anxiety***,*** and alcohol use disorder***?	

The second notable finding of our measure development process was regarding the scaling and the phrasing as questions rather than statements. Although the measure was originally designed as statements, we found that in piloting that phrasing as questions was easier for both providers and clients to understand (e.g., we changed “Clients are satisfied with services…,” to “How satisfied are clients with services…?”). Study team members in Nepal and South Africa reported that this decreased social desirability bias, thus having less risk of respondents providing affirmative responses across items. In Chile, a high-income country with a 94.6% literacy rate compared to Nepal with a 68% literacy rate, respondents were comfortable with the statement format of items, which they commonly encounter in formal education. However, to make the measure as universally usable as possible, including in LMICs, we decided to use the question format. Based on our biweekly discussions, we then selected a 4-point response scale, which were agreed as easiest to understand and code: 0 = Not at all; 1 = A little; 2 = A moderate amount; 3 = A lot. To support accurate coding, we included three additional options that could be used when assessments were being implemented by assessors (rather than self-report): 7 = Respondent refused to answer; 8 = Respondent doesn’t know; and 9 = Not applicable.

Our third major finding revolved around item selection, or the relevance of items by site. Here we asked respondents whether items were applicable to the specific task-sharing mental health intervention at hand, thus enabling evaluation of which barriers and facilitators showed relevance, as well as which were shared across sites. To illustrate, Table 4 shows the BeFITS-MH items (provider version) across all six core domains (including optional and omnibus items) rated by applicability (i.e., relevance) to the implemented task-sharing program at each of the three sites. Two main findings emerged. First, all the required items (and all the optional items except for two, Items 4.4 and 4.5), were rated as “applicable” by at least one site, thus indicating relevance of the vast majority of the identified constructs to task-sharing. This finding held true even though sites varied in the number of total items rated as “not applicable” (among sites, Chile rated 2 total items, South Africa rated 6 total items, and Nepal rated 11 total items as “not applicable”; of note, no omnibus item was rated as “not applicable” by any site). Second, common relevance of items across sites identified aspects that could be considered core components of barriers and facilitators. This emerged most clearly in Domain 4, “Provider Contextual Congruence”, where the task-sharing provider’s age (4.1), gender (4.2), being from the same community (4.3) and caste/ethnicity (4.6) were rated as relevant by all sites; conversely, optional items of provider’s social status (4.4) and religion (4.5) were rated as not relevant by all sites. These two items were rated as “not applicable” due to the perceived social inappropriateness of commenting upon some personal characteristics of the task-sharing provider that was expressed by respondents in South Africa and Chile. In Nepal, given the overlap of identity markers in this context (e.g., social status [4.4], religion [4.5], and caste/ethnicity [4.6]), only the item assessing provider’s caste/ethnicity [4.6] was retained. While items 4.4 and 4.5 were judged as not applicable to our three sites, we retained these items for testing in future locales. Similarly, in Domain 5, “Provider Accessibility and Availability”, the ease of talking to (5.1), availability of (5.2), and ease of contacting (5.3) the task-sharing provider were rated as relevant by all sites; conversely, optional items of regularly attending (5.4) and being on time for (5.5) the task-sharing service were rated as “not applicable” by one or more sites. These items were rated as not relevant because there were not different times for task-sharing and “standard” clinical services (i.e., the two were fully integrated) per the task-sharing programs delivered in Nepal and South Africa.

**Table 4:** Standard and Site-specific BeFITS-MH provider items

		Inclusion of BeFITS-MH Items			
Item #	Abbreviated Item Description	Standard	SMhINT	OTCH	RESHAPE
***Domain 1: Provider Role Fit***					
1.1	Able to provide service	✓	✓	✓	✓
1.2	Help clients participate in service	✓	**X**	✓	**X**
1.3	Preference for other provider	✓	✓	✓	✓
1	Domain 1 Omnibus Item	✓	✓	✓	✓
***Domain 2: Client Satisfaction***					
2.1	Care targets client problems	✓	✓	✓	✓
2.2	Helpfulness of care	✓	✓	✓	✓
2.3	Recommend care to others	✓	✓	✓	**X**
2	Domain 2 Omnibus Item	✓	✓	✓	✓
***Domain 3: Provider Competence***					
3.1	Understand client needs	✓	✓	✓	✓
3.2	Sympathize with client	✓	✓	✓	✓
3.3	Improve client’s knowledge of other MH programs	✓	✓	✓	**X**
3.4	Talk to clients in understandable way	✓	✓	✓	✓
3.5	Make service fit client’s needs	✓	✓	✓	**X**
3	Domain 3 Omnibus Item	✓	✓	✓	✓
***Domain 4: Provider Contextual Congruence***					
4.1	Provider’s age	✓	✓	✓	✓
4.2	Provider’s gender	✓	✓	✓	✓
4.3	Provider from the same community	✓	✓	✓	✓
4.4	Provider’s social status	Optional	**X**	**X**	**X**
4.5	Provider's religion	Optional	**X**	**X**	**X**
4.6	Provider’s caste/ethnicity	✓	✓	✓	✓
4	Domain 4 Omnibus Item	✓	✓	✓	✓
***Domain 5: Provider Accessibility & Availability***					
5.1	Easy to talk to provider	✓	✓	✓	✓
5.2	Availability of provider	✓	✓	✓	✓
5.3	Ease of contacting provider	✓	✓	✓	✓
5.4	Regularly attending task-sharing service	Optional	**X**	✓	**X**
5.5	On time for task-sharing service	Optional	✓	✓	**X**
5	Domain 5 Omnibus Item	✓	✓	✓	✓
***Domain 6: Client Support Systems***					
6.1	Family support of clients	✓	✓	✓	✓
6.2	Friends support of clients	✓	✓	✓	✓
6.3	Community members support	✓	✓	✓	**X**
6.4	Other healthcare providers support	✓	✓	✓	✓
6.5	Community leaders support	Optional	**X**	✓	**X**
6.6	Religious leaders support	Optional	**X**	✓	**X**
6	Domain 6 Omnibus Item	✓	✓	✓	✓

Check mark= applicable; X= not applicable

The final BeFITS-MH measure has two versions – one for Clients and one for Providers – assessing factors across seven core and two optional domains. The core domains include: (i) Client Satisfaction; (ii) Client Support Systems; (iii) Provider Role Fit; (iv) Provider Competence; (v) Provider Contextual Congruence; (vi) Provider Accessibility and Availability; and (vii) Provider Support Systems. The optional domains, (viii) Program Fit and (ix) Stigma, are hypothesized to be important to the successful implementation of task-sharing mental health interventions but are not specifically about the task-sharing strategy itself. Most domains include both required and optional items, and all domains include an omnibus item; the optional items can be used by implementers if determined to be appropriate for the local context. A summary of the BeFITS-MH domains and examples of each domain’s omnibus question is presented in Table 5. (The full BeFITS-MH measure is included in Additional File 1).

**Table 5:**
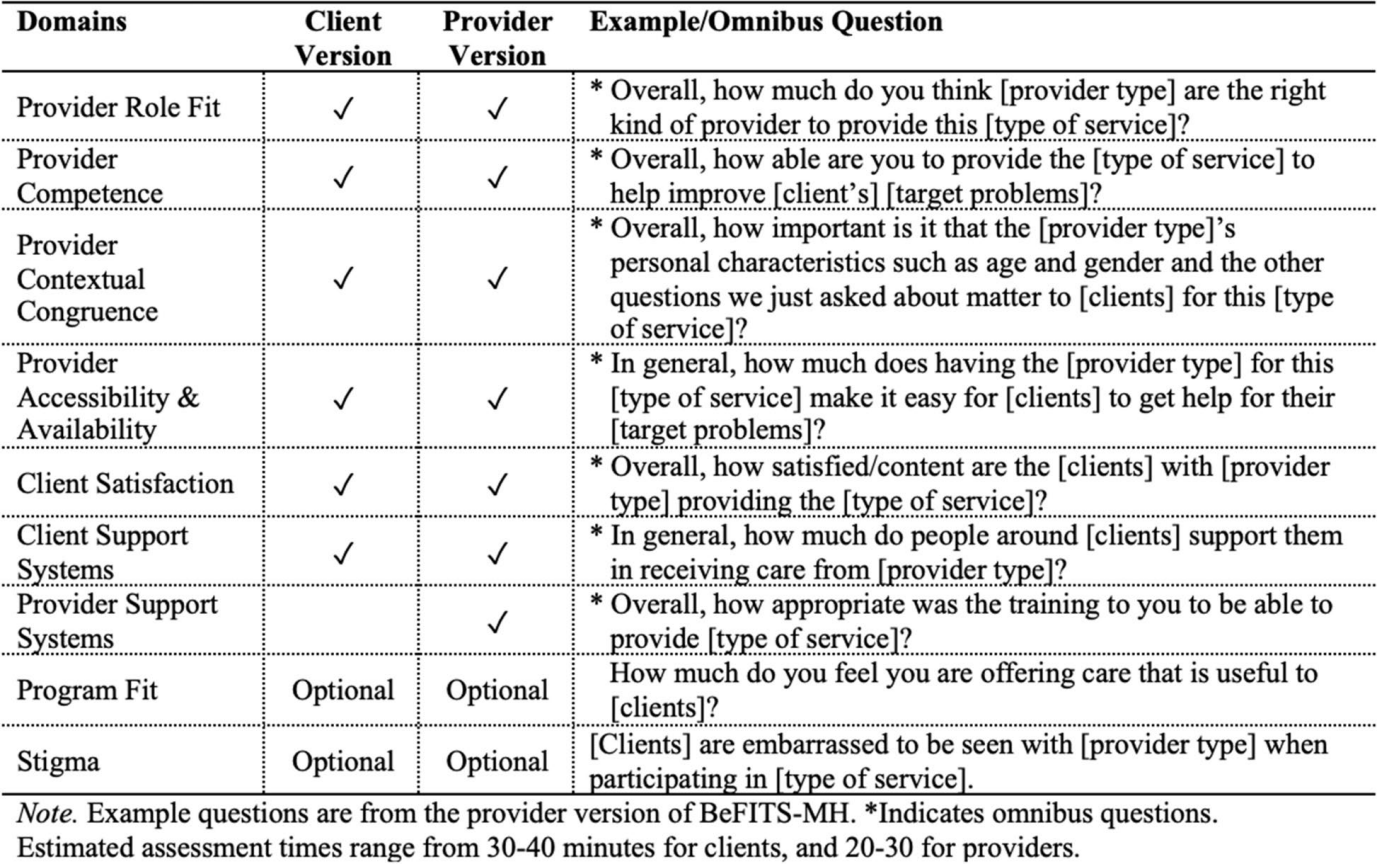
Description of BeFITS-MH domains

### Identification and assessment of key implementation outcomes to enhance utility

In the pilot testing of the three standard IS outcome measures (AIM, IAM, FIM) [45] the provider versions were deemed translatable and comprehensible by the task-sharing providers. However, in the Nepal and South Africa contexts, within each scale many of the items had the same translation in the local languages. For example, for the Intervention Appropriateness Measure (IAM), items of “seems fitting”, “seems suitable”, and “seems like a good match” all had the same terminology in Nepali; similarly, within the Feasibility of Intervention Measure (FIM), the items of “seems implementable”, “seems possible”, and “seems doable”, also were all translated with the exact or very similar wording. Across all sites, the versions of the three IS measures that we adapted for client respondents were deemed repetitive and difficult for service users to respond to. Because service users generally do not have experience with other mental health services, they were unable to compare and contrast their current service or provider with other experiences, and thus many reported that they did not understand how to respond. When asked to compare the different measures, both providers and clients found the tailored nature of the BeFITS-MH items easier to understand and respond to.

From the site-specific stakeholder discussions to identify indicators and assessment methods for the implementation outcomes (of acceptability, appropriateness, and feasibility) to enhance utility, four common categories of indicators emerged across all three outcomes: (I) uptake/adoption of the task-sharing mental health program by client, provider, and facility; (II) effectiveness/impact of the program in terms of the client health outcomes and the capability of providers to deliver more effective and relevant services; (III) ability to design and implement the program with oftentimes limited clinical resources and (IV) stigma-related issues (see Table 6).

**Table 6:** Example indicators of implementation outcomes stratified by main themes and level of measurement

Level of measurement	Implementation Outcomes		
	Acceptability	Appropriateness	Feasibility
	**Theme 1: Uptake/Adoption**		
*Client*	Adherence [Chile, OTCH] Number of follow up visits and referral slips [Nepal, RESHAPE]	Recommending services to others [Nepal, RESHAPE]	Satisfaction with services and the professionals providing those services [Chile, OTCH] Willingness to follow the program [Chile, OTCH] How well the program is adopted by users [Chile, OTCH]
*Provider*	**--**	Willingness to use and follow the program [Chile, OTCH]	**--**
*Facility/* *Clinic*	**--**	**--**	Adaptation and integration of services into the existing program [South Africa, SMhINT] Program is delivered as planned [South Africa, SMhINT]
	**Theme 2: Effectiveness/Impact**		
*Client*	Perceived usefulness of the program [Chile, OTCH] Number of clients referred to counseling who actually go (South Africa, SMhiNT)	Perception of program’s helpfulness for client’s health issues and other aspects of life [Chile, OTCH] Improvements in outcomes [Nepal, RESHAPE]	Reduction in symptoms [South Africa, SMhINT] Program helps patients with health needs [Chile, OTCH]
*Provider*	Value assigned to the program as an opportunity to grow professionally [Chile, OTCH] Ability of provider to identify client’s ‘pressing issue’ (South Africa, SMhiNT)	Seeing improvements in patients [Chile, OTCH]	**--**
	**Theme 3: Resource Constraints**		
*Facility/* *Clinic*	Number of providers, availability of physical space, availability of time to be trained [Chile, OTCH]	Adequate space and time [Chile, OTCH] Support from headquarters [Chile, OTCH]	Sufficient staff, availability of Counselors, inadequate physical space [South Africa, SMhINT] Medical roster identifying number of available health personnel [Nepal, RESHAPE]
	**Theme 4: Stigma**		
*Client*	Preference for separate mental health counseling and HIV counseling rooms [South Africa, SMhINT] Issues of privacy, safety, confidentiality [South Africa, SMhINT]	**--**	**--**
*Provider*	Preference for ‘Counselor’ vs. ‘Mental Health Counselor’ title [South Africa, SMhINT] Handling of sensitive data [Nepal, RESHAPE]	Measuring attitudes towards the mental health services [South Africa, SMhINT]	Sense that peer providers, by modeling recovery and reducing stigma, can enhance patient wellbeing [ South Africa, SMhINT]
*Facility/* *Clinic*	**--**	Facility has the space and resources for confidential information sharing between clients and providers [Nepal, RESHAPE]	**--**

The first set of indicators and assessment methods, evaluation of uptake/adoption at the client level, included indicators such as numbers of referrals, successful initiation of services, completed sessions, and follow up visits. Some stakeholders suggested that uptake/adoption at the provider level could be assessed by measuring factors such as provider’s willingness to use and follow the task sharing program, or whether services were provided as intended. At the facility/organization level, an understanding of whether and to what extent the task sharing program has been implemented (e.g., fidelity) or program components integrated into the organization would indicate program adoption. Notably, these uptake/adoption indicators were listed as ways to assess all three outcomes of acceptability, appropriateness, and feasibility, and by stakeholders across all three study sites.

The second set of indicators and assessment methods revolved around the effectiveness and impact of the task-sharing intervention. Most stakeholders mentioned indicators of program effectiveness for the clients, which included measurements such as improvements in client outcomes (e.g., symptom scores per standardized measures), or users’ and providers’ perceived ‘usefulness’ or ‘helpfulness’ of the specific task-sharing program in addressing clients’ health outcomes and other needs. Some stakeholders also noted assessing implementation outcomes in terms of the impact of the task-sharing intervention on providers’ professional development (e.g., the value providers assign to the program as an opportunity to grow professionally and expand their skillsets by providing effective services).

Issues related to resource constraints were identified as the third set of indicators and assessment methods, although these factors were most frequently mentioned with regard to feasibility. Stakeholders from all three sites highlighted clinical resource considerations such as having adequate measures, sufficient personnel and space, and resources to address patient needs in the context of frequently restricted resources. However, we were unable to ascertain specific indicators related to the task-sharing program’s ability to balance its activities with existing resource constraints.

Finally, stigma-related factors were identified as influential to all three implementation outcomes and to clients, providers, and health systems levels. Stigma was identified in relation to issues of confidentiality (e.g., whether facilities had space for confidential information sharing between clients and health providers; and that designated rooms [e.g., for mental health counseling] did not compromise client confidentiality by inadvertently identifying individuals as having a mental health condition). Related to task-sharing specifically, stakeholders in South Africa noted that they preferred to see task-sharing providers who were referred to generally as “counselors” rather than “mental health counselors”, and that peer providers in particular (i.e., persons with the illness [HIV] status themselves who are modeling recovery) were better suited to help patients overcome internalized stigma and effectively address their mental health problems.

## Discussion

This case study presents our process of developing and enhancing utility for a pragmatic IS measure with comprehensive items (i.e., content validity) and promising predictive properties (i.e., construct validity) as well as provides a detailed and nuanced description [34] for researchers and program implementers to identify and address barriers to the initiation, implementation, and sustainability of task-sharing mental health programs across three global contexts. This development process, where we employed a collaborative, multi-country, multi-stakeholder approach, can serve as a valuable case example for other teams developing IS measures, and provide support for considering content validity, contextual relevance (i.e., linguistic, cultural, and contextual adaptation), and pragmatic utility as key factors in the process of developing and enhancing validity for IS measures. In particular, we believe the concurrent adaptation and piloting across programs and global sites with multiple stakeholders from each site contributed novel strategies to standard measurement approaches. A core lesson that emerged is that targeting implementation measures towards actionable domains that could predict pragmatic markers of utility (e.g., effectiveness of an intervention) per program implementers’ preferences may generate implementation measures with greater content validity [6], relevance, and utility [8].

The development of the BeFITS-MH measure was guided by IS frameworks, including the CFIR and TDF, to capture generalizable IS constructs, and developed to be sufficiently targeted and brief to support pragmatic and sustainable use in task-sharing programs to support adaptation and quality improvement. Rigorous content validity was established through elucidation of barriers and facilitators to task-sharing mental health concepts using qualitative data from: (I) task-sharing mental health interventions previously conducted in three global sites; and (II) a systematic review, followed by review by an expert modified Delphi panel. The final BeFITS-MH domains each include 3–4 individual items and a single omnibus question; once validated, the measure could be as brief as 7 items if only the omnibus questions are used.

In the process of enhancing the future utility of the BeFITS-MH measure, the pilot testing and stakeholder discussions illustrated the perceived overlapping nature of the IS outcomes of acceptability, appropriateness, and feasibility. The stakeholders in particular provided feedback that many indicators and assessments can fit across multiple implementation outcomes. For example, indicators of uptake/adoption and effectiveness/impact fell across all three constructs of acceptability, appropriateness, and feasibility. These results suggest that what stakeholders value in terms of signaling useful implementation outcomes may not fit the traditional academic approach to treating these implementation outcomes as discrete, thus indicating a potential limitation of relying solely on these IS outcome measures. Results also serve to underscore the importance of stakeholder engagement at every stage of research [55], especially throughout the process of developing contextually-responsive, pragmatic [8], and valid [6] IS measures that have utility in applied global settings. Instead, conducting concurrent pilot testing and stakeholder analyses, such as what we have done in this study, may result in a validation process and measure that have greater content validity, meaning, and usefulness for program implementers.

The BeFITS-MH piloting activities, which were concurrently conducted in all three global study sites to maximize content validity, resulted in a measure that can be used in multiple countries and in different health delivery contexts. This is distinct from the typical approach of piloting and validating measures in a successive manner (i.e., site-by-site, context-by-context), and we believe that this simultaneous adaptation enables an advance from standard measurement approaches. Via this concurrent piloting and group translation approach, we were able to develop a harmonized measure that leveraged learnings from all sites simultaneously. Of note, all the required items (and nearly all the optional items) showed relevance to at least one site, indicating that our six identified BeFITS-MH domains were useful in assessing barriers and facilitators to task-sharing overall. Further, the BeFITS-MH domain of “Provider Contextual Congruence” showed congruence across all three sites where certain aspects of the task-sharing provider’s personal characteristics (i.e., age, gender, being from the same community, and ethnicity) were viewed as relevant, but other aspects such as the provider’s social status and religion were considered optional. These results illustrate that while each of our six identified BeFITS-MH domains appear universally relevant to task-sharing mental health interventions, the items that make up specific BeFITS-MH domains can vary by cultural context, what is being done in the task-sharing mental health intervention, and by the nature of the help provided. Finalizing item content while accounting for contextual variations in constructs (and translations) across the three contexts not only strengthened the measure’s potential global relevance and validity but exemplifies how measure development can, and should, align with the core tenet of “Rigor and Relevance” [56], ensuring the creation of rigorously developed, locally-adapted, and flexible tools for global implementation research.

In order to evaluate the ability of the BeFITS-MH measure to accurately assess the key implementation outcomes of acceptability, appropriateness and feasibility during future validity testing, we piloted three frequently used implementation outcome measures (AIM, IAM, and FIM) and identified the provider versions as useful, albeit with considerable limitations because of the idiomatic and redundant nature of the terminology when translated into local languages. The client versions were deemed neither comprehensible nor applicable. Because these three measures were designed initially for program implementers and higher-level systems administrators, the limited experience with mental health services of any kind among populations in LMICs, and their lack of familiarity with what alternative mental health services ‘should’ or ‘could’ look like, limited the validity and utility of these measures for the client level of measurement. Moreover, most prior studies with these measures in LMICs have been limited to English language versions of the measures and usage only among providers [46, 47].

Several limitations require noting in this case study to develop the BeFITS-MH measure. The tension between developing a measure that can be ‘universal’ and one that retains ‘location specific’ properties was present throughout the process and may prove illustrative for other study teams developing IS measures for global use. This tension was first exemplified in the discussion around item format (question vs. statement). Harmonizing across languages and program types resulted in creation of spaces in each question for projects to enter their own program-specific terminology for the intervention and provider type. On the one hand, this allowed needed project specificity in adapting the measure to fit how programs and providers are defined locally; on the other hand, this may also complicate comparisons between sites where programs and providers differ. In terms of conducting the BeFITS-MH measure piloting and stakeholder discussion fieldwork, the COVID-19 pandemic limited the number of assessments that could be completed. Nevertheless, each study site was able to obtain provider and systems-level stakeholder feedback in the translation and adaptation stages and when obtaining pilot data from providers and service users.

The process presented in this case study was done in part to prepare for the larger BeFITS-MH validation study, in which the BeFITS-MH measure is being embedded in each of the three study site’s longitudinal data collection procedures. A persistent challenge during the development and piloting process was the identification of appropriate indicators for validity testing. The piloting results raise the challenge of using measures such as the AIM, IAM, and FIM [45]. Given that these IS measures had limited comprehensibility and items were often interpreted as redundant, these measures were determined to be not optimal as measures for future construct validity testing. Further, the stakeholder discussions raised challenges in identifying the type and extent of administrative data available within the studies (i.e., uptake data) to operationalize implementation outcomes, and instead emphasized the importance of incorporating preferred indicators that are of clearer utility to program implementers (e.g., uptake/adoption; effectiveness of a task-sharing intervention). Discussions with site co-investigators are ongoing to identify available and appropriate indicators of implementation outcomes to support testing the future predictive validity of the BeFITS-MH measure, and whether other appropriate IS measures exist that could suit our purpose.

## Conclusion

A key goal of this case study was to describe the process of developing an IS measure that can be pragmatically useful across multiple diverse global settings with a range of different task-sharing mental health interventions. The challenges that we faced (e.g., identifying accurate terminology for key concepts in each locale, harmonizing translation across sites, identifying appropriate implementation outcomes and indicators for these for validity testing), and the rigorous strategies that we employed to address such challenges, can serve as a rich case description for other implementation research projects [34]. We believe this case study provides a roadmap for other research teams seeking to develop IS measures and locate appropriate measures by which to conduct validity testing, and to those who wish to maximize the local relevance, utility, and impact of their measures while ensuring global applicability.

The development of the BeFITS-MH measure is based on the need to improve identification of actionable factors that may enhance or impede uptake of mental health services delivered using task-sharing strategies. Identifying such factors will lead to more appropriately targeted systems-level interventions that, we hope, will support future scale up and sustainability of these evidence-based interventions and ultimately reduce the mental health treatment gap for populations around the globe.

## Supplementary Information

Supplementary Material 1. Full BeFITS-MH measure - Client and Provider Versions.

Supplementary Material 2. BeFITS-MH Delphi Questionnaires – Round 1 and Round 2.

## Data Availability

The datasets generated and/or analyzed during the current study are not publicly available but are available from the corresponding author on reasonable request.

## Abbreviations

AIM: Acceptability of Intervention Measure
BeFITS-MH: Barriers and Facilitators in Implementation of Task-Sharing in Mental Health services
CFIR: Consolidated Framework for Implementation Research
COSIMPO: Collaborative Shared care to Improve Psychosis Outcome
COVID-19: Coronavirus-19
CTI-TS: Critical Time Intervention-Task Shifting
EBP: Evidence based practice
FEP: First-Episode Psychosis
FIM: Feasibility of Intervention Measure
HIV: Human Immunodeficiency Virus
IAM: Intervention Appropriateness Measure
IAU: Intervention as Usual
IS: Implementation science
LMICs: Low- and middle-income countries
mhGAP-IG: Mental Health Gap Action Programme - Intervention Guide
Mphil: Master of Philosophy
NIMH: National Institute of Mental Health
NYC: New York City
OTCH: OnTrack Chile
PI: Principal Investigator
RE-AIM: Reach, Effectiveness, Adoption, Implementation, Maintenance framework
RESHAPE: Reducing Stigma among Healthcare Providers
SMhINT: Southern African Research Consortium for Mental Health Integration
TDF: Theoretical Domains Framework
THP-P: Thinking Healthy Program-Peer delivered

## Footnotes

1 The Shared Research Project included data from three studies: (1) the Critical Time Intervention-Task Shifting (CTI-TS) trial in Chile and Brazil; (2) the Thinking Healthy Program-Peer delivered (THP-P) project in India; and (3) the COllaborative Shared care to IMprove Psychosis Outcome (COSIMPO) study in Nigeria.
