# Supplementary material for: A case study of the development of a valid and pragmatic implementation science measure: the Barriers and Facilitators in Implementation of Task-Sharing Mental Health interventions (BeFITS-MH) measure
Source: BMC Health Serv Res. 2024 Nov 6;24:1352. doi: 10.1186/s12913-024-11783-6 (PMC11539761; doi:10.1186/s12913-024-11783-6)
Supplement: Supplementary file 1 — Supplementary Material 1. Full BeFITS-MH measure - Client and Provider Versions. [file 12913_2024_11783_MOESM1_ESM.docx]

| **Additional File 1. Full BeFITS-MH measure - Client and Provider Versions** | | | |
| --- | --- | --- | --- |
|  | **Client Version** | | **Provider Version** |
| Intro | The purpose of this survey is to ask you some questions about your experience participating in [PROGRAM], which involves [TYPE OF SERVICES] delivered by [PROVIDER TYPE] to help with [TARGET PROBLEM]. | | |
|  | When we ask about [PROVIDER TYPE], we are asking your opinions about the type of provider who is currently/has recently been providing you with the [TYPE OF SERVICE]; we understand that you may only be able to speak about your experiences with the [PROVIDER TYPE] that you have interacted with. | When we ask about [PROVIDER TYPE], we are asking your opinions about the type of provider who is providing the [TYPE OF SERVICE], although you might only be able to speak about your experiences with the [CLIENTS] that you have interacted with in the last 3 months. | |
| Scale | For each of the following questions, please select the number that best represents your opinion:  0=Not at all, 1=A little; 2=A moderate amount; 3=A lot  7=Refused to Answer; 8=Don't know; 9=Not Applicable | | |
| **1** | **DOMAIN: Provider Role Fit** | |  |
| 1.1 | How much is the [provider type] able to provide this [type of service]? | | How much are you able to provide this [type of service]? |
| 1.2 | How much is the [provider type] able to help you participate in this [type of service]? | | How much are you to help the [clients] participate in this [type of service]? |
| 1.3 | This [type of service] is currently being provided by [provider type]. How much better would it be if a [different provider type] provided this [type of service]? | | This [type of service] is currently being provided by [provider type]. How much better would it be if a [different provider type] provided this [type of service]? |
| **1*** | **Overall, how much do you think [provider type] is the right kind of provider to provide this [type of service]?** | | **Overall, how much do you think [provider type] are the right kind of provider to provide this [type of service]?** |
| **2** | **DOMAIN: Client Satisfaction** | | |
| 2.1 | How much is the [provider type] able to provide the care that you need for [target problem]? | | How much able are you to provide the care that [clients] need for [target problem]? |
| 2.2 | How helpful to you is the care provided by [provider type]? | | How helpful to the [clients] is the care that you provide? |
| 2.3 | How likely are you to recommend the [provider type] to help other people who have [target problems]? | | How likely are [clients] to recommend [provider type] to other people who have [target problems]? |
| **2*** | **Overall, how satisfied/content are you with [provider type] providing this [type of service]?** | | **Overall, how satisfied/content are the [clients] with [provider type] providing the [type of service]?** |
| **3** | **DOMAIN: Provider Competence** | | |
| 3.1 | How well can the [provider type] understand your needs? | | How well can you understand [clients’] needs? |
| 3.2 | How well can the [provider type] sympathize with you? | | How well can you sympathize with the [clients]? |
| 3.3 | How much can the [provider type] improve your knowledge about other services or tools (such as other program, pamphlets) to help with your [target problems]? | | How much can you improve [clients’] knowledge about other services or tools (such as other program, pamphlets) to help with their [target problems]? |
| 3.4 | How well can the [provider type] talk with you in a way that is easy for you to understand? | | How well can you talk to [clients] in a way that is easy for them to understand? |
| 3.5 | How well can the [provider type] make the [type of service] fit your needs? | | How well can you make the [type of service] fit [client’s] needs? |
| **3*** | **Overall, how able is the [provider type] to provide the [type of service] that helps with your [target problems]?** | | **Overall, how able are you to provide the [type of service] that help to improve [client’s] [target problems]?** |
| **4** | **DOMAIN: Provider Contextual Congruence** | | |
| 4.1 | How important is the [provider type]’s age for this [type of service], for example the [provider type] being the same age as the [client], or being older than the [client]? | | How important to the [clients] is the [provider type]’s age for [this type of service], for example the [provider type] being the same age as the [client], or being older than the [client]? |
| 4.2 | How important is it for the [provider type] and the [clients] to be the same gender? | | How important to the [clients] is it for the [provider type] and the [clients] to be the same gender? |
| 4.3 | How important is it that the [provider type] be from the same communities as the [clients]? | | How important to the [clients] is it for the [provider type] to be from the same communities as the [clients] ? |
| 4.4+ | How important is the [provider type]’s social status/role in your community to provide this [type of service]? | | How important to the [clients] is the [provider type]’s social status/role in the community to provide this [type of service]? |
| 4.5+ | How important is it for the [provider type] and the [clients] to have the same religious beliefs? | | How important to the [clients] is it for the [provider type] and the [clients] to have the same religious beliefs? |
| 4.6+ | How important is it for the [provider type] and the [clients] to have the same cultural/ethnic/caste? | | How important to the [clients] is it for the [provider type] and the [clients] to have the same cultural/ethnic/caste background? |
| **4*** | **Overall, how important is it that the [provider type]’s personal characteristics such as age and gender and the other questions we just asked about matter to [clients] for this [type of service]?** | | **Overall, how important to the [clients] is it that the [provider type]’s characteristics such as age and gender and the other questions we just asked about matter to [clients] for this [type of service]?** |
| **5** | **DOMAIN: Provider Accessibility & Availability** | | |
| 5.1 | How easy is it for you talk to this [provider type] about [target problems]? | | How easy is it for [clients] to talk to [provider type] about [target problems]? |
| 5.2 | How available is the [provider type] for this [type of service]? | | How available are [provider type] to [clients] for this [type of service]? |
| 5.3 | How easy is it for you to contact the [provider type] for help during a crisis? | | How easy is it for [clients] to contact the [provider type] for help during a crisis? |
| 5.4+ | How regularly does the [provider type] attend the [type of service]? | | How regularly does the provider type] attend the [type of service]? |
| 5.5+ | How on-time/punctual is the [provider type] typically for the [type of service]? | | How on-time/punctual is the [provider type] typically for the [type of service]? |
| **5*** | **In general, how much does having the [provider type] for this [type of service] make it easy for you to get help for your [target problems]?** | | **In general, how much does having the [provider type] for this [type of service] make it easy for [clients] to get help for their [target problems]?** |
| **6** | **DOMAIN: Client Support Systems (will include “they don’t know” option)** | | |
| 6.1 | How much does your family support your receiving care from [provider type]? | | How much do [clients]’ families support them in receiving care from [provider type]? |
| 6.2 | How much do your friends support your receiving care from [provider type]? | | How much do [clients’] friends support them in receiving care from [provider type]? |
| 6.3 | How much do community members support your receiving care from [provider type]? | | How much do community members support [clients] in receiving care from [provider type]? |
| 6.4 | How much do your other healthcare providers support your receiving care from [provider type]? | | How much do [clients’] other healthcare providers support them in receiving care from [provider type]? |
| 6.5+ | How much do community leaders support your receiving care from [provider type]? | | How much do community leaders support [clients] in receiving care from [provider type]? |
| 6.6+ | How much do your religious leaders support your receiving care from [provider type]? | | How much do religious leaders support [clients] in receiving care from [provider type]? |
| **6*** | **In general, how much do people around you support you in receiving care from [provider type]?** | | **In general, how much do people around [clients] support them in receiving care from [provider type]?** |
| **7** |  | | **DOMAIN: Provider Support Systems** |
| 7.1 |  | | How much has the training provided you with the knowledge needed to provide [type of service]? |
| 7.2 |  | | How much has the training provided you with the skills needed to provide [type of service]? |
| 7.3 |  | | How much has the training helped you develop the confidence to provide [type of service]? |
| 7.4 |  | | How easy was it for you to understand the training materials (e.g., the Counsellor Training Manual, pamphlets, posters)? |
| 7.5+ |  | | How useful were the training materials (e.g., the Counsellor Training Manual, pamphlets, posters)? |
| **7T*** |  | | **Overall, how appropriate was the training to you to be able to provide [type of service]?** |
|  |  | | *In the following, "supervisor" refers to [SUPERVISOR] who (will) support you in providing [type of service] to [clients].* |
| 7.7 |  | | How easily can you get helpful advice and support from a supervisor when you have a problem with a [client]? |
| 7.8 |  | | How available is a supervisor to answer your questions and concerns? |
| 7.9 |  | | How helpful are the communications you have with a/the [supervisor] for this [type of service]? |
| 7.10 |  | | How well supported are you in providing [type of service] by the other healthcare providers around you? |
| **7S*** |  | | **Overall, how much does the supervision you receive help you to provide [type of service] better?** |
| **8+** | **DOMAIN: Program Fit** | | |
| 8.1 | How much do you feel that the [provider type] is offering care that is useful to you? | | How much do you feel you are offering care that is useful to [clients]? |
| 8.2 | How satisfied are you with the amount of time you meet with [provider type]? | | How satisfied are [clients] with the amount of time you meet with them? |
| 8.3 | How satisfied are you with how often you meet with the [provider type], for example, do you feel like you meet with the [provider type] enough? | | How satisfied are [clients] with how often you meet them, for example do they feel like they meet with you enough? |
| 8.4 | How easily are you able get to where you receive the [type of service] from the [provider type]? | | How easily can the [clients] get to where you provide the [type of service]? |
| 8.5 | How safe do you feel where you get the [type of service] from the [provider type]. | | How safe do the [clients] feel where you provide the [type of service]. |
| 8.6 | How private is the place where you get the [type of service] from the [provider type]? | | How private is the place where you provide the [type of service]? |
| **9+** | **DOMAIN: Stigma** | | |
|  | **Attitudes of Client**  *In this section, we will read some statements about your experience with [receiving/providing] [type of service]. Please select the number that best represents your opinion:*  0=Not at all, 1=A little; 2=A moderate amount; 3=A lot  7=Refused to Answer; 8=Don't know; 9=Not Applicable | | |
| 9A.1 | I am embarrassed to be seen with [provider type] when participating in [type of service]. | | [Clients] are embarrassed to be seen with [provider type] when participating in [type of service]. |
| 9A.2 | I do not think that getting treatment from [provider type] for the [target problems] is as important as getting treatment for my general/physical health problems (e.g., heart disease). | | [Clients] do not think that getting treatment from [provider type] for the [target problems] is as important as getting treatment for their general/physical health problems (e.g., heart disease). |
| 9A.3 | I feel less stigmatized/ looked down on to see a [provider type] instead of a [MH specialist]. | | [Clients] feel less stigmatized/looked down on to see a [provider type] like me instead of a [MH specialist]. |
| 9A.4 | I am embarrassed to tell my friends or family that I am receiving [this type of service] from [provider type]. | | [Clients] feel embarrassed to tell their friends or family that they are receiving [this type of service] from [provider type] like me. |
|  | **Attitudes about Clients**  *In this section, we use the word “Others” to mean people in your family, community members, community leaders, co-workers, etc.* | | |
| 9B.1 | Others may think people who use [type of service] are weak in character. | | Others may think [clients] who use [type of service] are weak in character. |
| 9B.2 | Others may think people who use [type of service] brought the [type of problem] on themselves. | | Others may think [clients] who use [type of service] brought the [type of problem] on themselves. |
| 9B.3 | Others may think people who use [type of service] are less worthy of help (or not as important to help) compared to people with other health problems. | | Others may think [clients] who use [type of service] are less worthy of help (or not as important to help) compared to people with other health problems. |
| 9B.4 | Others may think people who use [type of service] are not likely to recover from their condition. | | Others may think [clients] who use [type of service] are not likely to recover from their condition. |
| 9B.5 | Others would look down on me if they were to find I am receiving [type of service] from [provider type]. | | Others would look down on [clients] if they were to find out the [client] was receiving [this type of service] from [provider type] like me. |
| 9B.6 | Others would look down on my family if they were to find I am receiving [type of service] from [provider type]. | | Others would look down on [clients’] families if they were to find out the [client] was receiving [this type of service] from [provider type] like me. |
| 9B.7 | Others knowing that I receive [type of service] from [provider type] will be a barrier to participating in school/training, work or community activities. | | Others knowing that the client receives [type of service] from [provider type] would be a barrier to [clients’] participation in school/training, work or community activities. |
|  |  | | **Provider’s Experience** *As a reminder, please note that you do not have to answer any question that you feel uncomfortable with.* |
| 9C.1 |  | | [Clients] in [type of service] treat me with respect. |
| 9C.2 |  | | My colleagues look down on me because I deliver [type of service] to [clients]. |
| 9C.3 |  | | Providing [type of service] to [clients] will lead to opportunities to advance my career/provides me valuable professional skills. |
| 9C.4 |  | | My facility supervisor/manager [and the institution] do not encourage me to put time into this [type of service] for [clients]. |
| 9C.5 |  | | Being [provider type] is not a worthwhile use of my time. |
| 9C.6 + |  | | [Clients] in [type of service] respect me just as much as other healthcare workers. |
| 9C.7+ |  | | I am not compensated for delivering this [type of service] to [clients] in the same way that I am compensated for [other type of service – e.g., maternal health] |
| *We have reached the end of the survey.*  *Thank you very much for sharing your experiences and opinions with us.* | | | |
