# Supplementary material for: A case study of the development of a valid and pragmatic implementation science measure: the Barriers and Facilitators in Implementation of Task-Sharing Mental Health interventions (BeFITS-MH) measure
Source: BMC Health Serv Res. 2024 Nov 6;24:1352. doi: 10.1186/s12913-024-11783-6 (PMC11539761; doi:10.1186/s12913-024-11783-6)
Supplement: Supplementary file 2 — Supplementary Material 2. BeFITS-MH Delphi Questionnaires – Round 1 and Round 2. [file 12913_2024_11783_MOESM2_ESM.pdf]

BeFITS-MH Delphi Round 1

The purpose of this round of Delphi is to elicit your feedback on the major domains that the BeFITS-MH measure should include, and the items that should be included in each domain.

The BeFITS-MH measure has 2 versions: Client and Provider.

The measure currently includes 6 proposed domains:
1. Provider Role Fit
2. Motivation & Satisfaction
3. Provider Competence
4. Contextual Congruence
5. Service Availability
6. Support Systems

We will first ask you about the relevance of the domains, then about the proposed items in each domain.

The link to the full measure with all the domains and the items will be included in the following sections.

* Indicates required question

Relevance of Domains

In this section, we would like your feedback on the relevance of each of the domains, from both the perspective of Clients as well as Providers.

You can access the full measure (version 1) here: https://bit.ly/3f5x4m7. (We recommend that you have the new browser side by side you can you refer to the full measure.)

The survey has 2 versions:
(A) Client
(B) Providers

There are currently 6 domains:

1. PROVIDER ROLE FIT:
Participants' understanding of the role of the task-sharing provider and whether the role fits with the intervention/program and clients' mental health needs.

2. MOTIVATION & SATISFACTION:
Participants' satisfaction with the task-sharing provider's services in terms of the structure of the services (e.g., number and duration of sessions) and participants' willingness to continue with the services.

3. PROVIDER COMPETENCE:
Task-sharing provider's ability to understand and address clients' mental health needs.

4. CONTEXTUAL CONGRUENCE:
Compatibility between clients' and task-sharing provider's personal and professional backgrounds.

5. SERVICE AVAILABILITY:
Extent to which task-sharing provider's services are available and accessible to the clients.

6. SUPPORT SYSTEMS:
This domain has 2 versions, Client and Provider/Supervisor.
For Clients, support systems refer to the extent of the support that Clients have from family and community members about their participation in the program.
For Providers, the support refers to the training and the supervision that was provided to the task-sharing provider to help carry out their role.

1. Based on the brief description of the domains above, and the items currently proposed for the domain, please rate the relevance of each domain -- from the perspective of the CLIENTS. (Link to full measure is here: https://bit.ly/3f5x4m7)

Mark only one oval per row.

	Low	Medium	High
**1. Provide Role Fit**	◯	◯	◯
**2. Motivation & Satisfaction**	◯	◯	◯
**3. Provider Competence**	◯	◯	◯
**4. Contextual Congruence**	◯	◯	◯
**5. Service Accessibility**	◯	◯	◯
**6. Support Systems**	◯	◯	◯

2. Please elaborate on why you selected each answer for the domains.

____

____

____

____

____

3. Based on the brief description of the domains above, and the items currently proposed for the domain, please rate the relevance of each domain -- from the perspective of the PROVIDERS. (Link to full measure is here: https://bit.ly/3f5x4m7)

Mark only one oval per row.

	Low	Medium	High
**1. Provide Role Fit**	◯	◯	◯
**2. Motivation & Satisfaction**	◯	◯	◯
**3. Provider Competence**	◯	◯	◯
**4. Contextual Congruence**	◯	◯	◯
**5. Service Accessibility**	◯	◯	◯
**6. Support Systems**	◯	◯	◯

4. Please elaborate on why you selected each answer for the domains.

____

____

____

____

____

Domain 1: Provider Role Fit

This domain refers to participants' understanding of the role of the task-sharing provider and whether the role fits with the intervention/program and clients' mental health needs.

Each item has parallel versions, with wording adapted for either the Clients or the Providers.
For example, in 1.1:
Client version: "I have a good understanding of what the role of the [provider] is."
Provider version: "I have a good understanding of what my role is."

5. Does each of the following items fit in this domain? Please evaluate the items for their core constructs across the client/provider versions.

**1: Provider Role Fit**		
	Client Version	***Provider Version***
**1.1**	I have a good understanding of what the role of the [provider] is.	I have a good understanding of what my role is.
**1.2**	It makes sense to have [providers] with this role.	It makes sense to have [providers] with this role.
**1.3**	I think the role of the [provider] is important.	I think [this role] is important.
**1.4**	The [provider] is offering services that are useful to me.	I am offering services that are useful to [clients].
**1.5**	The [provider] has the appropriate educational/professional training for the role.	I have the appropriate educational/professional training for the role.
**1.6**	It would be better if a different type of [provider] delivers the [program].	It would be better if a different type of provider delivers the [program].
**1.7**	I think the [provider] is the right kind of person to give this program.	I think I am the right kind of person to give this program.

Mark only one oval per row.

	Yes	No	Not Sure
**1.1**	◯	◯	◯
**1.2**	◯	◯	◯
**1.3**	◯	◯	◯
**1.4**	◯	◯	◯
**1.5**	◯	◯	◯
**1.6**	◯	◯	◯
**1.7**	◯	◯	◯

6. Please elaborate on your selections above. Feel free to suggest wording changes. If you have specific wording changes for certain items, please indicate which item (e.g., 1.1) and the version (Client/Provider).

7. What other important concepts or items do you think should be included in this domain?

8. What additional comments do you have about the domain?

Domain 2: Motivation and Satisfaction

This domain refers to participants' satisfaction with the task-sharing provider's services in terms of the structure of the services (e.g., number and duration of sessions) and participants' willingness to continue with the services.

Each item has parallel versions, with wording adapted for either the Clients or the Providers.
For example, in 2.1:
Client version: "Overall, I like the services I receive from the [provider]."
Provider version: "Overall, [clients] like the services they receive from me."

9. Does each of the following items fit in this domain? Please evaluate the items for their core constructs across the client/provider versions.

2: Motivation & Satisfaction		
	Client Version	***Provider Version***
**2.1**	Overall, I like the services I receive from the [provider].	Overall, [clients] like the services they receive from me.
**2.2**	The [provider] meets my expectations.	I meet the [client's] expectations.
**2.3**	The [provider] motivates me to attend [sessions].	I motivate [clients] to attend [sessions].
**2.4**	The [provider] helps improve my well-being.	I help improve [client's] well-being.
**2.5**	I learn important skills from the [provider].	I learn important skills from the [program].
**2.6**	I am satisfied with the number of visits I have with the [provider].	I am satisfied with the number of visits I have with [clients].
**2.7**	I am satisfied with the duration of the sessions with the [provider].	I am satisfied with the duration of the sessions with [clients].

Mark only one oval per row.

	Yes	No	Not Sure
**2.1**	◯	◯	◯
**2.2**	◯	◯	◯
**2.3**	◯	◯	◯
**2.4**	◯	◯	◯
**2.5**	◯	◯	◯
**2.6**	◯	◯	◯
**2.7**	◯	◯	◯

10. Please elaborate on your selections above. Feel free to suggest wording changes. If you have specific wording changes for certain items, please indicate which item (e.g., 2.2) and the version (Client/Provider).

____

____

____

____

____

11. What other important concepts or items do you think should be included in this domain?

12. What additional comments do you have about the domain?

Domain 3: Provider Competence

This domain refers to the task-sharing provider's ability to understand and address clients' mental health needs.

Each item has parallel versions, with wording adapted for either the Clients or the Providers.
For example, in 3.1:
Client version: "The [provider] has a good understanding of my needs."
Provider version: "I have a good understanding of [clients'] needs."

13. Does each of the following items fit in this domain? Please evaluate the items for their core constructs across the client/provider versions.

3. Provider Competence		
	Client Version	***Provider Version***
**3.1**	The [provider] has a good understanding of my needs.	I have a good understanding of [clients'] needs.
**3.2**	The [provider] can provide the support that I need.	I can provide the support that [clients] need.
**3.3**	The [provider] can relate to me.	I can relate to [clients].
**3.4**	The [provider] improves my knowledge about mental health.	I improve [clients'] knowledge about mental health.
**3.5**	The [provider] conducts sessions well.	I conduct sessions well.
**3.6**	The [provider] communicates with me in a clear manner.	I communicate with [clients] in a clear manner.
**3.7**	The [provider] conducts himself/herself professionally during sessions.	I conduct myself professionally during sessions.
**3.8**	The [provider] adjusts treatment approach to fit my needs.	I adjust treatment approach to fit [client's] needs.

Mark only one oval per row.

	Yes	No	Not Sure
**3.1**	⬭	⬭	⬭
**3.2**	⬭	⬭	⬭
**3.3**	⬭	⬭	⬭
**3.4**	⬭	⬭	⬭
**3.5**	⬭	⬭	⬭
**3.6**	⬭	⬭	⬭
**3.7**	⬭	⬭	⬭
**3.8**	⬭	⬭	⬭

14. Please elaborate on your selections above. Feel free to suggest wording changes. If you have specific wording changes for certain items, please indicate which item (e.g., 3.3) and the version (Client/Provider).

15. What other important concepts or items do you think should be included in this domain?

16. What additional comments do you have about the domain?

Domain 4: Contextual Congruence

This domain refers to the compatibility between clients' and task-sharing provider's personal and professional backgrounds.

Each item has parallel versions, with wording adapted for either the Clients or the Providers.
For example, in 4.1:
Client version: "I feel comfortable with the [provider's] background."
Provider version: "[Clients] feel comfortable with my background."

17. Does each of the following items fit in this domain? Please evaluate the items for their core constructs across the client/provider versions.

4: Contextual Congruence		
	Client Version	***Provider Version***
4.1	I feel comfortable with the [provider's] background.	[Clients] feel comfortable with my background.
4.2	The [provider's] background fits with my background and values.	My background fits with [client's] background and values.
4.3	The [provider's] background fits with my religious beliefs.	My background fits with [client's] religious beliefs.
4.4	The [provider's] background fits with my cultural values.	My background fits with [client's] cultural values.
4.5	The [provider's] background fits with the community norms and conditions.	My background fits with the community norms and conditions.
4.6	The [provider's] background fits with my beliefs about mental health services/treatment.	My background fits with [client's] beliefs about mental health services/treatment.
4.7	The [provider's] background allows him/her to understand the community issues affecting my mental health.	My background allows me to understand the community issues affecting [clients'] mental health.
4.8	The [provider] helps me understand program materials.	I help [clients] understand program materials.

Mark only one oval per row.

	Yes	No	Not Sure
**4.1**	⬭	⬭	⬭
**4.2**	⬭	⬭	⬭
**4.3**	⬭	⬭	⬭
**4.4**	⬭	⬭	⬭
**4.5**	⬭	⬭	⬭
**4.6**	⬭	⬭	⬭
**4.7**	⬭	⬭	⬭
**4.8**	⬭	⬭	⬭

18. Please elaborate on your selections above. Feel free to suggest wording changes. If you have specific wording changes for certain items, please indicate which item (e.g., 4.4) and the version (Client/Provider).

19. What other important concepts or items do you think should be included in this domain?

20. What additional comments do you have about the domain?

Domain 5: Service Availability

This domain refers to the extent to which task-sharing provider's services are available and accessible to the clients.

Each item has parallel versions, with wording adapted for either the Clients or the Providers.
For example, in 5.1:
Client version: "The sessions with [providers] are held in a convenient place."
Provider version: "The sessions with [clients] are held in a convenient place."

21. Does each of the following items fit in this domain? Please evaluate the items for their core constructs across the client/provider versions.

5. Service Availability		
	Client Version	*Provider Version*
5.1	The sessions with [providers] are held in a convenient place.	The sessions with [clients] are held in a convenient place.
5.2	The sessions with [providers] are held in a comfortable place.	The sessions with [clients] are held in a comfortable place.
5.3	It was difficult to schedule sessions with the [provider].	It was difficult to schedule sessions with [clients].
5.4	[Clients] can reach the [provider] easily.	[Clients] can reach me easily.
5.5	[Provider] is flexible when scheduling appointments.	I am flexible with [clients] when scheduling appointments.
5.6	[Provider] attended scheduled sessions on time.	I attend scheduled sessions on time.
5.7	I have a difficult time fitting [program activities] into my daily schedule.	I have a difficult time fitting [program activities] into my daily work schedule.

Mark only one oval per row.

	Yes	No	Not Sure
5.1	⬭	⬭	⬭
5.2	⬭	⬭	⬭
5.3	⬭	⬭	⬭
5.4	⬭	⬭	⬭
5.5	⬭	⬭	⬭
5.6	⬭	⬭	⬭
5.7	⬭	⬭	⬭

22. Please elaborate on your selections above. Feel free to suggest wording changes. If you have specific wording changes for certain items, please indicate which item (e.g., 5.5) and the version (Client/Provider).

____

____

____

____

____

23. What other important concepts or items do you think should be included in this domain?

24. What additional comments do you have about the domain?

Domain 6: Support Systems

This domain contains different items for Clients and Providers.

For Clients, support systems refer to the extent of the support that Clients have from family and community members about their participation in the program.

For Providers, the support refers to the training and the supervision that was provided to the task-sharing provider to help carry out their role.

25. 6A. Support Systems - Client: Does each of the following items fit in the domain? Please evaluate the items for their core constructs.

6A. Support Systems - Client	
6.1A	My family is comfortable with me receiving the services from the [provider].
6.2A	My family thinks participation in [program] with the [provider] improves my well-being.
6.3A	My family supports my participation in [program] with the [providers].
6.4A	My friends and neighbors know of my participation in [program] with the [providers].
6.5A	My friends and neighbors do not like my participation in [program] with the [providers].
6.6A	Some people whom I respect (e.g. religious/spiritual leaders, community leaders) do not like my participation in [program] with the [providers].
6.7A	I am unable to participate in some [sessions] with the [providers] because of transportation costs.
6.8A	I am unable to participate in some [sessions] with the [providers] because of other financial issues.

Mark only one oval per row.

	Yes	No	Not Sure
**6.1A**	◯	◯	◯
**6.2A**	◯	◯	◯
**6.3A**	◯	◯	◯
**6.4A**	◯	◯	◯
**6.5A**	◯	◯	◯
**6.6A**	◯	◯	◯
**6.7A**	◯	◯	◯
**6.8A**	◯	◯	◯

26. Please elaborate on your selections above (6A). Feel free to suggest wording changes. If you have specific wording changes for certain items, please indicate which item (e.g., 6.6A).

27. What other important concepts or items do you think should be included in this domain (6A)?

28. What additional comments do you have about the domain?

29. 6B. Support Systems - Provider: Does each of the following items fit in the domain? Please evaluate the items for their core constructs.

6B. Support Systems - Provider	
6.1B	Training provided me with the skills needed to [deliver the program].
6.2B	Training provided me with the knowledge needed to [deliver the program].
6.3B	Training helped me develop the confidence to [deliver the program.]
6.4B	The language used during training was easy to understand.
6.5B	The training materials were useful.
6.6B	Overall, I like the supervision sessions.
6.7B	Supervision provided helpful feedback.
6.8B	[Supervisor] was available to answer my questions and concerns.

Mark only one oval per row.

	Yes	No	Not Sure
6.1B			
6.2B			
6.3B			
6.4B			
6.5B			
6.6B			
6.7B			
6.8B			

30. Please elaborate on your selections above (6B). Feel free to suggest wording changes. If you have specific wording changes for certain items, please indicate which item (e.g., 6.6B).

31. What other important concepts or items do you think should be included in this domain (6B)?

32. What additional comments do you have about the domain?

Additional Domains and Comments

33. What other domains do you think should be added to the BeFITS-MH measure in order to capture the modifiable barriers and facilitators of implementing task-sharing mental health interventions? What are some example items?

34. What other comments do you have for this version of the BeFITS-MH measure?

Thank you!

Thank you for completing the survey! We will share the results of this Delphi round with you and communicate regarding the next steps shortly.

For internal record-keeping, please provide your name and e-mail address.

35. Please enter your full name (Last, First). *

36. Please enter your e-mail address.

This content is neither created nor endorsed by Google.

Google Forms

BeFITS-MH Delphi (Round 2)

The purpose of this round of Delphi (round 2) is to elicit your feedback on the revised BeFITS-MH measure, Version 2.

The revised BeFITS-MH measure still has two versions: Client and Provider.
The BeFITS-MH v2 has 6 main domains, an additional domain for providers only (domain 7), and a potential optional domain (domain 8).

1. Provider Role Fit
2. Motivation & Satisfaction
3. Provider Competence
4. Contextual Congruence
5. Accessibility & Availability
6. Support Systems
7. Training & Supervision (for task-sharing providers only)
8. Program Fit (optional)

Note:
* indicates items that might be considered omnibus questions
+ indicates optional items

Please find the link to the BeFITS-MH Version 2 here: https://bit.ly/BeFITS-MHv2
For definitions of the domains, please click here: https://bit.ly/BeFITS-MHdomainsIO.

You can also look at the side-by-side comparison of the changes made between the two versions here: https://bit.ly/BeFITS-MHcomparev1v2

1. Email *

____

Item Fit

In this section, please indicate whether you think each item should be included in the specified domain.

Link for BeFITS-MH Version 2: https://bit.ly/BeFITS-MHv2
Link for definitions of the domains: https://bit.ly/BeFITS-MHdomainsIO.

2. DOMAIN 1: Do you agree that the item should be included in the domain?

	Client Version	Provider Version
1	Provider Role Fit	
1.1	I have a good understanding of what the role of [provider type] is.	I have a good understanding of what my role is.
1.2	I think the role of [provider type] is relevant to my mental health.	I think the role of [provider type] is relevant to [clients'] mental health.
1.3	I think the [provider type] can provide [this type of service].	I think I can provide this type of service -- [service].
1.4+	It would be better if [a different provider type] provides [service].	It would be better if [a different provider type] provides [service].
1*	**Overall, I think [provider type] is the right type of provider to offer [service].**	**Overall, I think [provider type] is the right type of provider to offer [service].**

Mark only one oval per row.

	No, do not include	Yes, as is	Yes, need changes
**1.1**	⬭	⬭	⬭
**1.2**	⬭	⬭	⬭
**1.3**	⬭	⬭	⬭
**1.4+**	⬭	⬭	⬭
**1* (omnibus)**	⬭	⬭	⬭

3. (D1) Please elaborate on your responses above. If you selected "Yes, need changes," include your suggested changes. Feel free to include additional items.

____

____

____

____

____

4. DOMAIN 2: Do you agree that the item should be included in the domain?

	Client Version	*Provider Version*
2	Satisfaction	
2.1	The [provider type] meets my expectations.	I meet the [client's] expectations.
2.2	The [provider type] motivates me to attend [sessions].	I motivate [clients] to attend [sessions].
2.3	The [provider type] helps improve my mental health.	I help improve [client's] mental health.
2*	**Overall, I like [services] I receive from [provider type].**	**Overall, [clients] like [services] they receive from me.**

Mark only one oval per row.

	No, do not include	Yes, as is	Yes, need changes
**2.1**	◯	◯	◯
**2.2**	◯	◯	◯
**2.3**	◯	◯	◯
**2* (omnibus)**	◯	◯	◯

5. (D2) Please elaborate on your responses above. If you selected "Yes, need changes," include your suggested changes. Feel free to include additional items.

____

____

____

____

____

6. DOMAIN 3: Do you agree that the item should be included in the domain?

	Client Version	*Provider Version*
3	Provider Competence	
3.1	The [provider type] has a good understanding of my mental health needs.	I have a good understanding of [clients'] mental health needs.
3.2	The [provider type] can relate to me.	I can relate to [clients].
3.3	The [provider type] improves my knowledge about mental health.	I improve [clients'] knowledge about mental health.
3.4	The [provider type] conducts sessions well.	I conduct sessions well.
3.5	The [provider type] talks to me in a way that makes me feel comfortable getting [this type of service].	I talk to [clients] in a way that make them feel comfortable getting [this type of service].
3.6	The [provider type] adjusts treatment approach to fit my needs.	I adjust treatment approach to fit [client's] needs.
3.7	The [provider type] helps me learn important skills to improve my mental health.	I help [clients] learn important skills to improve their mental health.
3.8+	The [provider type] helps me understand program materials.	I help [clients] understand program materials.
**3***	**Overall, the [provider type] can provide the mental health support that I need.**	**Overall, I can provide the mental health support that [clients] need.**

Mark only one oval per row.

	No, do not include	Yes, as is	Yes, need changes
**3.1**	◯	◯	◯
**3.2**	◯	◯	◯
**3.3**	◯	◯	◯
**3.4**	◯	◯	◯
**3.5**	◯	◯	◯
**3.6**	◯	◯	◯
**3.7**	◯	◯	◯
**3.8+**	◯	◯	◯
**3* (omnibus)**	◯	◯	◯

7. (D3) Please elaborate on your responses above. If you selected "Yes, need changes," include your suggested changes. Feel free to include additional items.

8. DOMAIN 4: Do you agree that the item should be included in the domain?

	Client Version	Provider Version
4	Contextual Congruence	
4.1	I am comfortable with my [provider type's] age.	[Clients] are comfortable with my age.
4.2	I am comfortable with my [provider type's] gender.	[Clients] are comfortable with my gender.
4.3	I am comfortable with my [provider type's] religious beliefs.	[Clients] are comfortable with my religious beliefs.
4.4	I am comfortable with my [provider type's] cultural/ethnic background.	[Clients] are comfortable with my cultural/ethnic background.
**4***	**The [provider type] understands what is happening in the community that affects my mental health.**	**I understand what is happening in the community that affects [clients'] mental health.**

Mark only one oval per row.

	No, do not include	Yes, as is	Yes, need changes
**4.1**	⬭	⬭	⬭
**4.2**	⬭	⬭	⬭
**4.3**	⬭	⬭	⬭
**4.4**	⬭	⬭	⬭
**4* (omnibus)**	⬭	⬭	⬭

9. (D4) Please elaborate on your responses above. If you selected "Yes, need changes," include your suggested changes. Feel free to include additional items.

10. DOMAIN 5: Do you agree that the item should be included in the domain?

	Client Version	Provider Version
5	Accessibility & Availability	
5.1	The [provider type] is approachable.	I am approachable to [clients].
5.2	I can schedule sessions with the [provider type] easily if needed.	[Clients] can schedule sessions with me easily if needed.
5.3	I can contact the [provider type] easily if needed.	[Clients] can contact me easily if needed.
5.4+	The [provider type] attends the scheduled sessions.	I attend the scheduled sessions.
5.5+	The [provider type] attends sessions on time.	I attend sessions on time.
5*	**In general, the [provider type] is available at a time that works for me.**	**In general, I make myself available at a time that works for [clients].**

Mark only one oval per row.

	No, do not include	Yes, as is	Yes, need changes
**5.1**	⬭	⬭	⬭
**5.2**	⬭	⬭	⬭
**5.3**	⬭	⬭	⬭
**5.4+**	⬭	⬭	⬭
**5.5+**	⬭	⬭	⬭
**5* (omnibus)**	⬭	⬭	⬭

11. (D5) Please elaborate on your responses above. If you selected "Yes, need changes," include your suggested changes. Feel free to include additional items.

12. DOMAIN 6: Do you agree that the item should be included in the domain? Note: [community leaders] can be unspecified, or can be specified to refer to important figures in the clients' community, such as religious leaders.

	Client Version	Provider Version
6	Support Systems	
6.1	My family supports me [getting this type of service] from [provider type].	My family supports me [providing this type of service] to [clients].
6.2	My friends and neighbors support me [getting this type of service] from [provider type].	My friends and neighbors support me [providing this type of service] to [clients].
6.3	My [community leaders] support me [getting this type of service] from [provider type].	My [community leaders] support me [providing this type of service] to [clients].
6*	**In general, people around me support me [getting this type of service] from [provider type].**	**In general, people around me support me [providing this type of service] to [clients].**

Mark only one oval per row.

	No, do not include	Yes, as is	Yes, need changes
**6.1**	⬭	⬭	⬭
**6.2**	⬭	⬭	⬭
**6.3**	⬭	⬭	⬭
**6* (omnibus)**	⬭	⬭	⬭

13. (D6) Please elaborate on your responses above. If you selected "Yes, need changes," include your suggested changes. Feel free to include additional items.

14. DOMAIN 7 (Providers only): Do you agree that the item should be included in the domain?

7	Training and Supervision (Providers only)
7.1	Training provided me with the skills needed to [deliver the service].
7.2	Training provided me with the knowledge needed to [deliver the service].
7.3	Training helped me develop the confidence to [deliver the service.]
7.4	The language used during training was easy to understand.
7.5	The training materials were useful.
**7T***	**Overall, I have the appropriate training for the role.**
7.7	When I have a problem with a client, I can get helpful advice and support from [supervisor].
7.8	[Supervisor] was available to answer my questions and concerns.
7.9	The approach to supervision will work for me in the long-term.
**7S***	**Overall, I like the supervision sessions.**

Mark only one oval per row.

	No, do not include	Yes, as is	Yes, need changes
**7.1**	◯	◯	◯
**7.2**	◯	◯	◯
**7.3**	◯	◯	◯
**7.4**	◯	◯	◯
**7.5**	◯	◯	◯
**7T* (omnibus: training)**	◯	◯	◯
**7.7**	◯	◯	◯
**7.8**	◯	◯	◯
**7.9**	◯	◯	◯
**7S* (omnibus: supervision)**	◯	◯	◯

15. (D7) Please elaborate on your responses above. If you selected "Yes, need changes," include your suggested changes. Feel free to include additional items.

Implementation Outcomes

16. Please select the implementation outcome(s) that the domain corresponds to. You can select more than one outcome for each domain. You can also select the outcome only if it somewhat fits. Please note in the open-ended text box if you have any in-depth feedback.

Definitions of Domains

Domain	Definition
1. Provider Role Fit	Participants' understanding of the role of the task-sharing provider and whether the role fits with the clients' mental health needs.
2. Motivation & Satisfaction	Participants' satisfaction with the task-sharing provider's services in terms of the structure of the services (e.g., number and duration of sessions) and participants' willingness to continue with the services
3. Provider Competence	Task-sharing provider's ability to understand and address clients' mental health needs
4. Contextual Congruence	Compatibility between clients' and task-sharing provider's backgrounds (i.e., age, gender, race/ethnicity, religion)
5. Accessibility & Availability	Extent to which task-sharing provider's services are available and accessible to the clients
6. Support Systems	Extent to which family, friends and neighbors, and community members support clients' and task-sharing providers' participation in the program

Definitions of Implementation Outcomes
(from Proctor et al. 2011)

Implementation Outcome	Definition
Acceptability	the perception among implementation stakeholders that a given treatment, service, practice, or innovation is agreeable, palatable, or satisfactory
Appropriateness	the perceived fit, relevance, or compatibility of the innovation or evidence-based practice for a given practice setting, provider, or consumer; and/or perceived fit of the innovation to address a particular issue or problem
Feasibility	the extent to which a new treatment, or an innovation, can be successfully used or carried out within a given agency or setting

Check all that apply.

	Acceptability	Appropriateness	Feasibility	None of these	Unsure
**D1: Provider Role Fit**	☐	☐	☐	☐	☐
**D2: Satisfaction**	☐	☐	☐	☐	☐
D3: Provider Competence					

D3: Provider Competence	☐	☐	☐	☐	☐
D4: Contextual Congruence	☐	☐	☐	☐	☐
D5: Accessibility & Availability	☐	☐	☐	☐	☐
D6: Support Systems	☐	☐	☐	☐	☐
D7: Training & Supervision (provider only)	☐	☐	☐	☐	☐

17. (IO) Provide your comments on your responses above.

____

____

____

____

____

Domain 8: Program Fit

The items in this domain are thought to be potentially important/relevant items that specific programs might be interested in assessing.

"Program Fit" items

	Client Version	Provider Version
8+	Program Fit	
8.1	The [provider type] is offering services that are useful to me.	I am offering services that are useful to [clients].
8.2	I am satisfied with the number of sessions I have with [provider type].	I am satisfied with the number of sessionsI have with [clients].
8.3	I am satisfied with the duration of the sessions with [provider type].	I am satisfied with the duration of the sessions with [clients].
8.4	The [provider type] conducts sessions in a place that I can get to easily.	I conduct sessions in a place that [clients] can get to easily.
8.5	The [provider type] conducts sessions in a place that I feel safe being in.	I conduct sessions in a place that [clients] feel safe being in.
8.6	The [provider type] conducts sessions in a place that can ensure confidentiality.	I conduct sessions in a place that can ensure confidentiality.
8.7	I am unable to participate in some sessions with [provider type] because of transportation costs.	n/a
8.8	I am unable to participate in some sessions with [provider type] because of other financial issues.	n/a

18. (8i1) Do you agree with separating these items into a separate "Program Fit" domain?

Mark only one oval.

- Yes
- No
- Unsure

19. (8i2) Please explain your answer above.

20. (8ii) To what extent do you think any of the items fit into the main BeFITS-MH measure itself, which is intended to assess the barriers and facilitators of the task-sharing strategy?

21. (8iii) Would any of the items be useful/relevant to your/certain task-sharing programs?

Conclusion

22. Do you have any other comments or suggestions about the current BeFITS-MH measure?

This content is neither created nor endorsed by Google.

Google Forms
